# Rare recurrence pattern after complete response to chemotherapy in a patient with rectal cancer: a case report

**DOI:** 10.1186/s40792-024-01913-x

**Published:** 2024-05-08

**Authors:** Masatoshi Nomura, Mitsuyoshi Tei, Takayoshi Goto, Masataka Hirano, Kentaro Nishida, Soichiro Mori, Yukihiro Yoshikawa, Koki Tamai, Takuya Hamakawa, Daisuke Takiuchi, Masanori Tsujie, Yusuke Akamaru

**Affiliations:** 1 Department of Gastroenterological Surgery, Osaka Rosai Hospital, Nagasone 1179-3, Kita Ward, Sakai, Osaka 591-8025 Japan; 2 Department of Pathology, Osaka Rosai Hospital, Sakai, Osaka 591-8025 Japan

**Keywords:** Colorectal cancer, Metastasis, Small intestine, Skin, Bladder

## Abstract

**Background:**

Colorectal cancer** (**CRC) often metastasizes to the liver, lungs, lymph nodes, and peritoneum but rarely to the bladder, small intestine, and skin. We here report the rare metastasis of anal cancer in the left bladder wall, followed by metastases to the small intestine and skin, after abdominoperineal resection and left lateral lymph node dissection with chemotherapy in a patient with clinician Stage IVa disease.

**Case presentation:**

A 66-year-old man presented with 1-month history of bloody stool and anal pain and diagnosed with clinical Stage IVa anal cancer with lymph node and liver metastases (cT3, N3 [#263L], M1a [H1]). Systemic chemotherapy led to clinical complete response (CR) for the liver metastasis and clinical near-CR for the primary tumor. Robot-assisted laparoscopic perineal rectal resection and left-sided lymph node dissection were performed. Computed tomography during 18-month postoperative follow-up identified a mass in the left bladder wall, which was biopsied with transurethral resection, was confirmed as recurrent anal cancer by histopathologic evaluation. After two cycles of systemic chemotherapy, partial resection of the small intestine was performed due to bowel obstruction not responding to conservative therapy. The histopathologic evaluation revealed lymphogenous invasion of the muscularis mucosa and subserosa of all sections. Ten months after the first surgery for bowel obstruction and two months before another surgery for obstruction of the small intestine, skin nodules extending from the lower abdomen to the thighs were observed. The histopathologic evaluation of the skin biopsy specimen collected at the time of surgery for small bowel obstructions led to the diagnosis of skin metastasis of anal cancer. Although panitumumab was administered after surgery, the patient died seven months after the diagnosis of skin metastasis.

**Conclusions:**

This case illustrates the rare presentation of clinical Stage IVa anal cancer metastasizing to the bladder wall, small intestine, and skin several years after CR to chemotherapy.

## Background

Colorectal cancer (CRC), one of the most prevalent cancer types and the second most common cause of cancer death worldwide in 2020 [1]. CRC often metastasizes to the liver, lungs, lymph nodes, and peritoneum [2] and rarely to the bladder, small intestine, and skin [3–5]. Metastasis to the small intestine most commonly occurs via local invasion and rarely via the lymphogenous and hematogenous routes [6].

Here, we present the rare case of a patient with clinical Stage IVa anal cancer who experienced recurrence after achieving complete response (CR) to chemotherapy following abdominoperineal resection and left lateral lymph node dissection.

## Case presentation

**Fig. 1:**
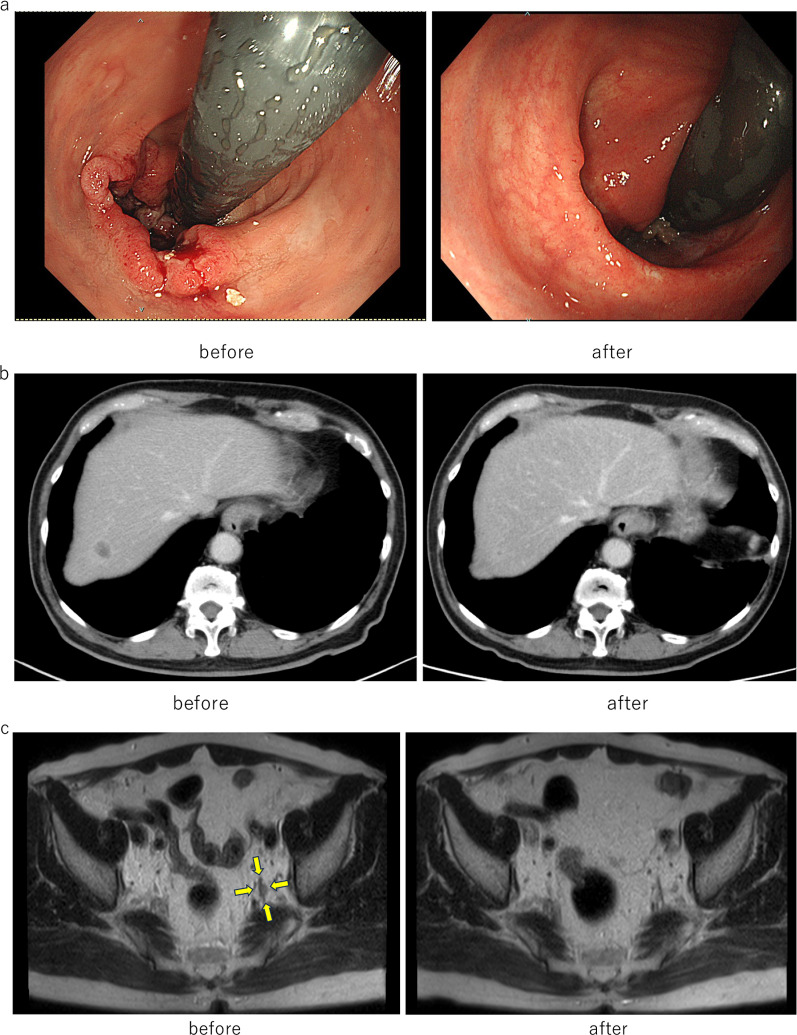
**a** Shrinkage of rectal cancer with chemotherapy, indicating near complete response, by lower gastrointestinal endoscopy. **b** Complete response of the single liver metastasis in S6 following chemotherapy. **c** Magnetic resonance imaging showing suspicious metastasis in lymph node #263L, which has shrunk with chemotherapy and is not detected on imaging

A 66-year-old man presented with 1-month history of bloody stool and anal pain. His history included hypertension, hyperlipidemia, hyperuricemia, traffic trauma, and postoperative right inguinal hernia. He had no history of smoking. The histopathologic evaluation of the biopsy specimen obtained with colonoscopy led to the diagnosis of moderately differentiated tubular adenocarcinoma of the anal canal. The diagnosis was Stage IVa with lymph node and liver metastases (T3, N3 [#263L], M1a [H1]) (Fig. 1a–c) based on evaluation with computed tomography and magnetic resonance imaging [7]. RAS and BRAF gene status were wild type. MSI test was performed postrecurrence and was negative.

After six cycles of systemic chemotherapy with 5-fluorouracil, leucovorin, oxaliplatin, and irinotecan (FOLFOXIRI) plus bevacizumab, clinical CR and clinical near-CR were detected in the liver metastatic lesion and the primary tumor, respectively, and surgery was planned (Fig. 1a–c). Robot-assisted laparoscopic perineal rectal amputation and left-sided lymph node dissection were performed with no postoperative complications. Pathological diagnosis was ypT0, N0, M0 ypStage0. Postoperative adjuvant chemotherapy was not administered, and the patient was evaluated every 3 months in the outpatient clinic.

**Fig. 2:**
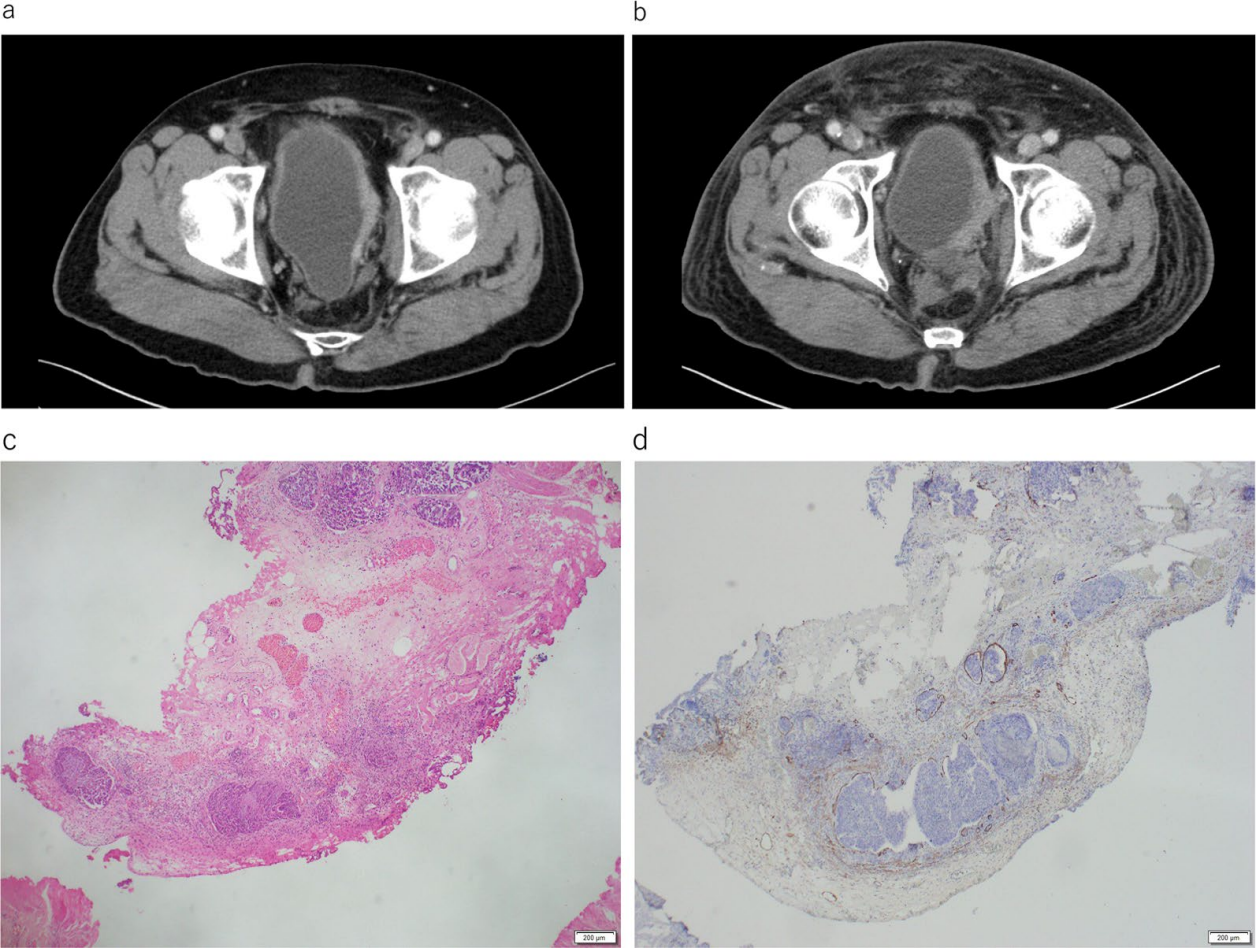
**a** Thickening of the left bladder wall observed in computed tomography images obtained at 1-year postoperative follow-up. **b** Computed tomography at 18-month postoperative follow-up showing further thickening of the bladder wall. **c** Hematoxylin/eosin staining shows tumor cells in the bladder wall. **d** Immunohistochemical staining for CD40 shows extensive lymphatic invasion of tumor cells in the bladder wall

**Fig. 3:**
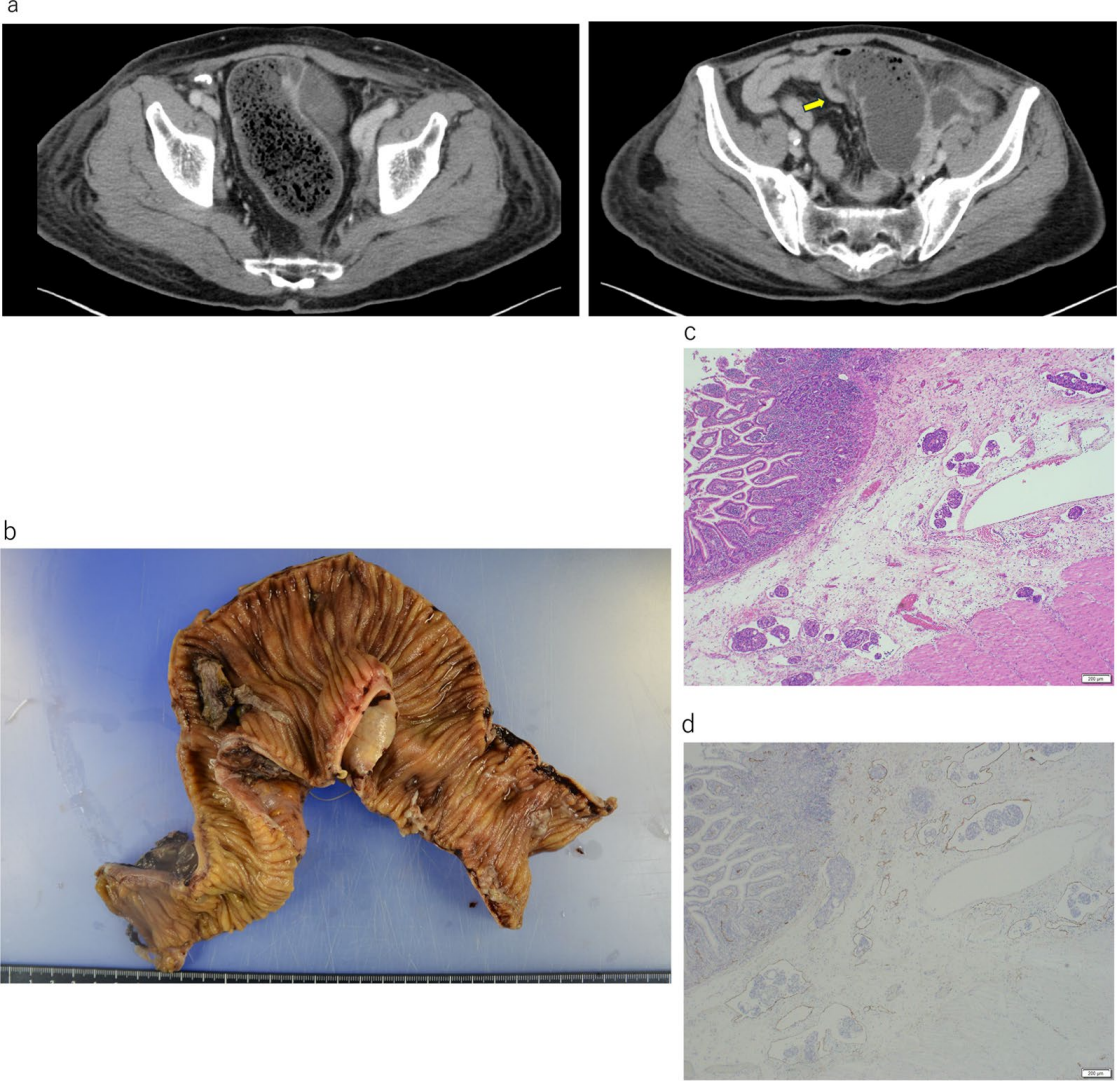
**a** Small intestinal obstruction due to adhesions detected by computed tomography. **b** Mucosal lesions are not observed in the excised small intestinal specimen. **c** Hematoxylin/eosin staining showing lymphatic invasion of the submucosa and muscularis mucosa without obvious tumor formation. **d** Immunohistochemical staining for CD40 shows tumor cells only in the lymphatic vessels

Computed tomography performed during 1-year postoperative follow-up revealed thickening of the left bladder wall (Fig. 2a), requiring cystoscopy, which revealed no significant findings. Repeat cystoscopy was performed 6 months later because of the worsening of the left bladder wall thickening detected by computed tomography (Fig. 2b). The deformity in the left bladder wall, which was biopsied by transurethral resection, was diagnosed as metastasis of anal cancer. The histopathologic examination of the resected tissue revealed that the tumor cells had invaded the lymphatic vessels of the bladder. The tumor cells did not invade venous vessels, and histology of this tumor was similar to rectal cancer, which is a moderately differentiated tubular adenocarcinoma (Fig. 2c, d). After two cycles of systemic chemotherapy with FOLFOXIRI plus bevacizumab, partial resection of the small intestine was performed because of the difficulty in resolving obstruction of the small intestine with conservative therapy (Fig. 3a). The evaluation of the resected small intestine revealed lymphogenous invasion of the muscularis mucosa and subserosa of all sections, although tumor formation in intestinal mucosa were not observed (Fig. 3b–d).

**Fig. 4:**
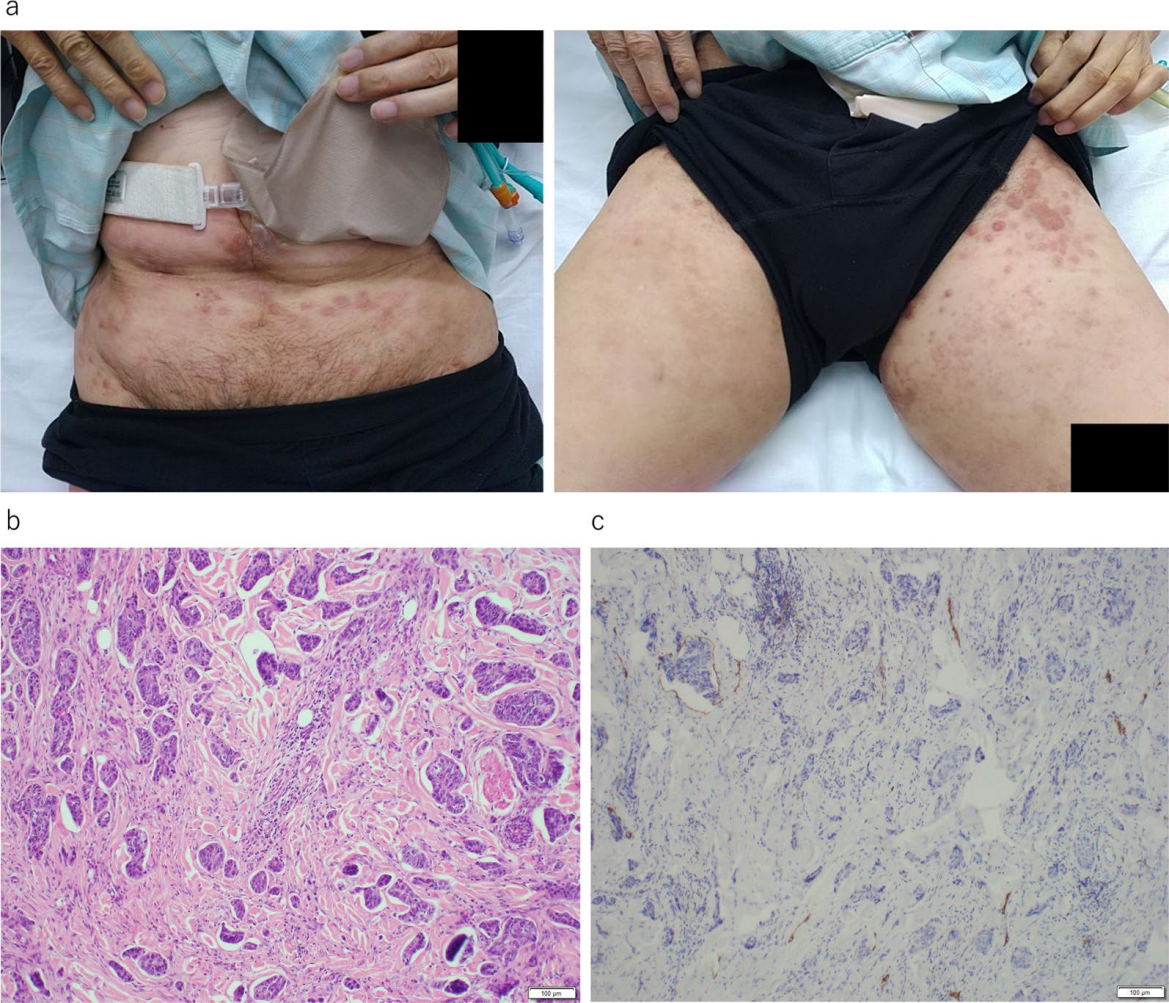
**a**–**c** Skin nodules are observed extending from the lower abdomen to the lower extremities. **d** Biopsy results show numerous tumor cells in subcutaneous lymphatic vessels

**Fig. 5:**
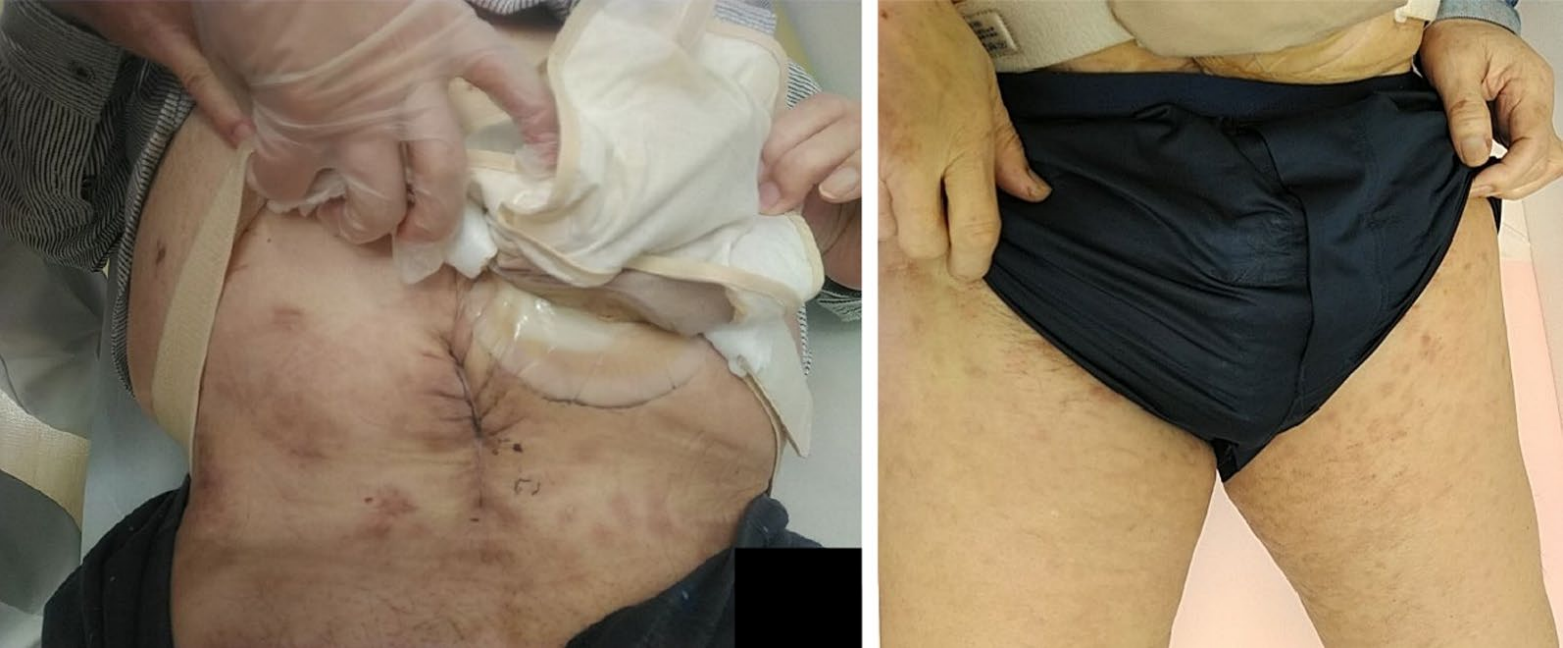
**a**–**c** Skin nodules tend to disappear after the administration of panitumumab

After excluding oxaliplatin, which had to be discontinued due to allergic reaction, the chemotherapy regimen was continued for approximately 10 months. However, ten months after the first surgery for small bowel obstruction, skin nodules extending from the lower abdomen to the thighs were observed (Fig. 4a); the nodules gradually worsened over few months. The histopathologic evaluation of the biopsy specimens of the nodules collected at the time of the second surgery for bowel obstruction led to the diagnosis of skin metastasis of anal cancer (Fig. 4b, c). Following the administration of panitumumab after surgery, the skin nodules appeared to get smaller (Fig. 5). However, four months after the diagnosis of skin metastasis, the patient developed lymphangitis carcinomatosa, discontinued chemotherapy, and died three months later.

## Discussions/conclusions

In the present case, the primary tumor was located in the front of the anal canal, tumor cells were found in left lateral lymph node #263, and one synchronous liver metastasis was detected. Therefore, the diagnosis before chemotherapy was Stage IVa (T3N3M1a). The histopathologic examination of the resected tissue revealed that chemotherapy resulted in pathological CR in the primary tumor and the left lateral lymph node; in addition, the left lateral lymph node #263 confirmed the scar of metastasis from the primary tumor. We predict that the primary tumor had already metastasized to the bladder from the left lateral lymph node prior to the initiation of systemic chemotherapy. In some patients with CRC, direct invasion to the bladder is observed. In these cases, the tumor in the bladder likely metastasized to the lateral lymph node on the same side because bladder cancer often metastasizes to lateral lymph nodes [8].

In the present case, the primary tumor and the liver metastasis had disappeared by pathologic and clinical evaluation. Despite the CR observed at these sites, the patient presented with bladder metastasis and the lymphogenous invasion in the muscularis propria and subserosa of the small intestine were observed in the specimens resected during surgery of bowel obstruction. The clinical diagnosis of tumors metastasizing to the small intestine via the hematogenous or lymphogenous route is challenging; in the present case, tumor cells were observed only in the lymphatic vessels and a metastatic mass was not found in the small intestine. The presence of tumor cells at multiple sites within the small intestinal wall layers and on both ends of the stump in the resected specimen support the presence of cancer in the remnant small intestine.

The diagnosis of small intestinal metastasis is often based on symptoms such as obstruction and bleeding; the present patient underwent emergency surgery due to small intestinal obstruction [9, 10]. However, the cause of obstruction was adhesions and not the tumor invasion of the lymphatic ducts, resembling occult metastasis. Metachronous small intestinal metastasis may be considered in cases where the cause of small intestinal obstruction after CRC surgery is not clear; however, such cases are rare [11]. Skin metastasis, which is rare in patients with CRC, might occur through lymphogenous spread from the small intestine or the bladder. The prognosis of CRC with only small intestinal metastasis is good in patients undergoing curative resection [9]. The metastatic pattern of such cases might be distinct from the pattern observed in the present case, such as the hematogenous spread.

The present patient did not develop lung or recurrent liver metastasis. However, he had pedal edema with weight gain and the skin metastasis occupied the lower abdomen and thighs, which might be a result of the blockage of the lymphatic circulation by primary tumor resection and chemotherapy. Some studies have reported that the resection of only the small intestinal or skin metastasis of CRC was associated with good prognosis [9, 12, 13]. However, lymphogenous invasion of the bladder, small intestine, and skin observed in the present case is very rarely reported. The conceivable route of metastasis is from the left lateral lymph node to the left bladder wall, with later spread to the small intestine and skin through the lymphogenous route. The overall survival time was 22 months from the bladder metastasis, 19 months from the lymphogenous invasion of the small intestine, and 7 months from the skin metastasis, reflecting the poor course and prognosis.

## Data Availability

All data generated or analyzed during this study are included in this published article.

## Abbreviations

CR: Complete response
CRC: Colorectal cancer
